# Supplementary material for: Single-cell phenotypic plasticity modulates social behavior in Dictyostelium discoideum
Source: iScience. 2023 May 2;26(6):106783. doi: 10.1016/j.isci.2023.106783 (PMC10206496; doi:10.1016/j.isci.2023.106783)
Supplement: Document S1. Figures S1–S7 [file mmc1.pdf]

iScience, Volume *26*

Supplemental information

Single-cell phenotypic plasticity modulates social behavior in *Dictyostelium discoideum*

Mathieu Forget, Sandrine Adiba, and Silvia De Monte

Single-cell phenotypic plasticity modulates social behaviour in *Dictyostelium discoideum* .

Mathieu Forget[1,2], Sandrine Adiba[1,*], Silvia De Monte[1,2,*],

1 Institut de Biologie de l'Ecole Normale Supérieure, Département de Biologie, Ecole Normale Supérieure, CNRS, INSERM, PSL Research University, Paris, France

2 Department of Evolutionary Theory, Max Planck Institute for Evolutionary Biology, Plőn, Germany

* These authors contributed equally to this work.

Lead contacts: Mathieu Forget (forget@bio.ens.psl.eu) and Silvia De Monte (demonte@evolbio.mpg.de)

Supplementary Information

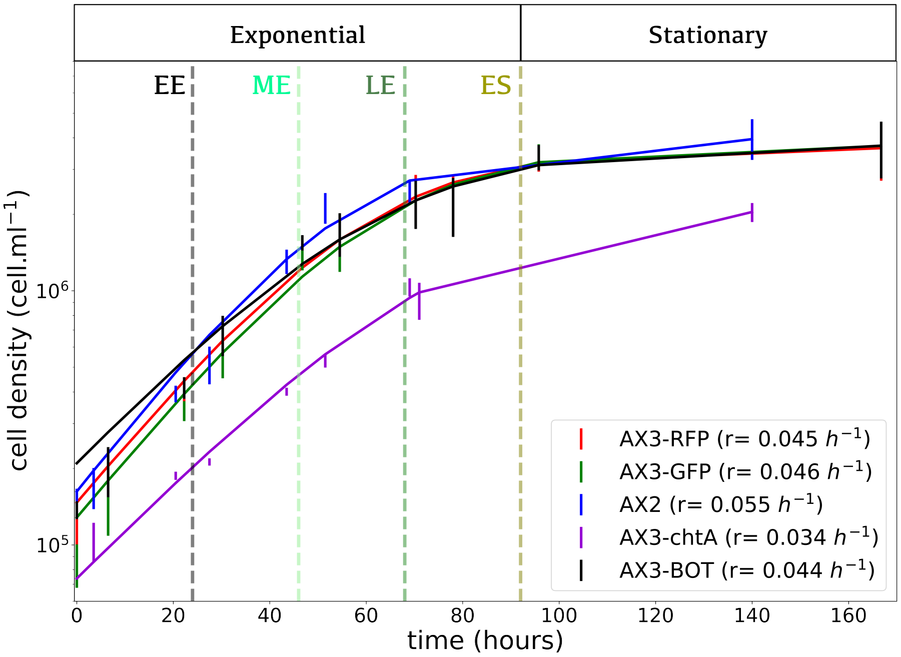

Figure S1: **Growth kinetics of the different strains used in the experiments (related to STAR Methods).** Cell density of cultures grown in 25 cm^2 flasks with 10 ml HL5 medium were assessed in triplicates using an hemocytometer. Before the beginning of the experiment, pre-cultures were prepared from frozen aliquots as described in the Methods. All strains had similar growth kinetics, but ChtA's initial density was slightly lower due to fluctuations at the moment of dilution. The exponential and stationary phases of growth are indicated, as well as the times of harvesting considered in chronochimeras (vertical dotted lines), as explained in the methods. The average net growth rate in exponential phase (computed by linear interpolation between 20 and 50 hours) is reported for all the strains.

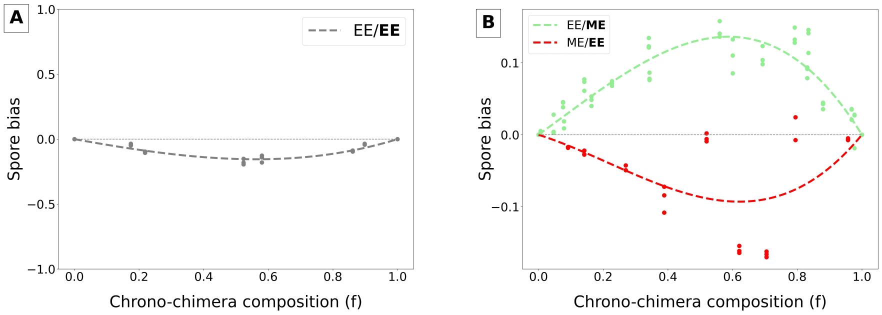

Figure S2: **A: AX3-GFP spore bias at the end of chimeric development with AX3-RFP cells (related to STAR Methods).** The two populations were grown in co-cultures for 24 hours prior to aggregation to ensure they are in the same growth phase at the onset of the social cycle. Transformation with different plasmids introduced a spore bias (consistent when the transformation was repeated). We used this frequency-dependent bias to correct the measures realized in isogenic chronochimeras (main text and Methods). **B: Check for consistency of spore bias measures.** Corrected spore bias measured in two types of 'chrono-chimeras', using the AX3-GFP as the reference population: the AX3-GFP population was harvested in ME phase and mixed with a EE AX3-RFP population (light green line), and vice-versa (red line). Spore biases are reversed when the fluorescent labels are swapped, validating the use of the frequency correction.

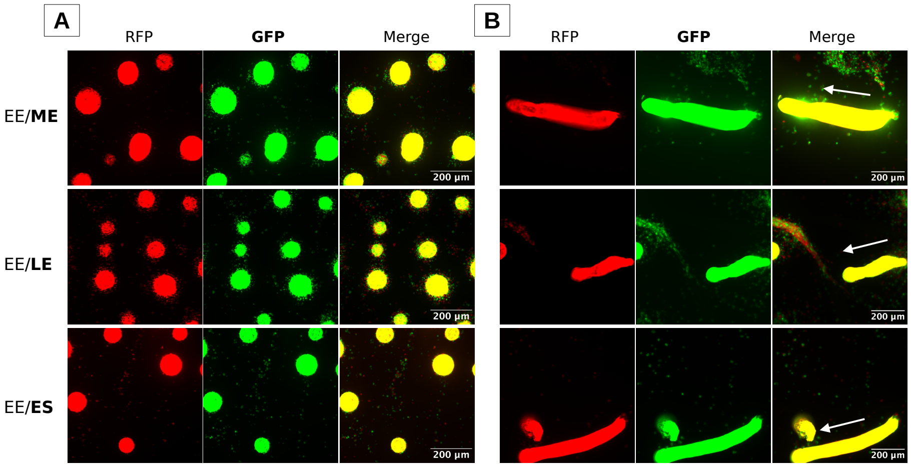

Figure S3: **Heterogeneity in populations growth phase at the onset of starvation does not translate into detectable cell sorting during *Dictyostelium* social cycle (related to Fig. 3).** AX3-RFP cells harvested in EE phase of the growth cycle are mixed in chimeras with AX3-GFP cells harvested either in ME (first row), LE (second row) or ES phase (third row). **A:** RFP and GFP-cell populations do not segregate during aggregation but rather form chimeric aggregates with no noticeable difference in composition between aggregates, nor evident spatial sorting within aggregates. **B:** RFP and GFP-cell populations do not show significant signs of sorting along the slug axis during its migration. The white arrow indicates the direction of slug migration. Notice that since all cells bear a fluorescent marker, the high density of the mound and slug stages make it impossible to distinguish single cells in the images.

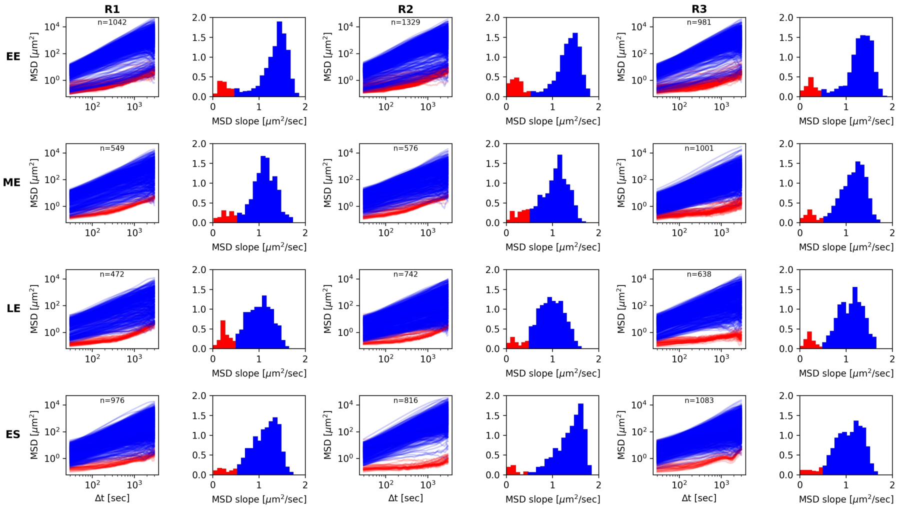

Figure S4: **Bimodal distribution of single-cell motility depends on growth phase (related to Fig. 5).** Individual mean square displacement of cells from populations harvested in EE, ME, LE and ES phase as a function of time lag (Δt). Cells were clustered into two classes based on the initial rate of increase of the MSD (slope of the log MSD vs log Δt, $\Delta t < 150$ secs, below or above the threshold value 0.5). As shown in Fig. 4 C, the proportion of cells belonging to the non-migrating class decreases in the course of vegetative growth.

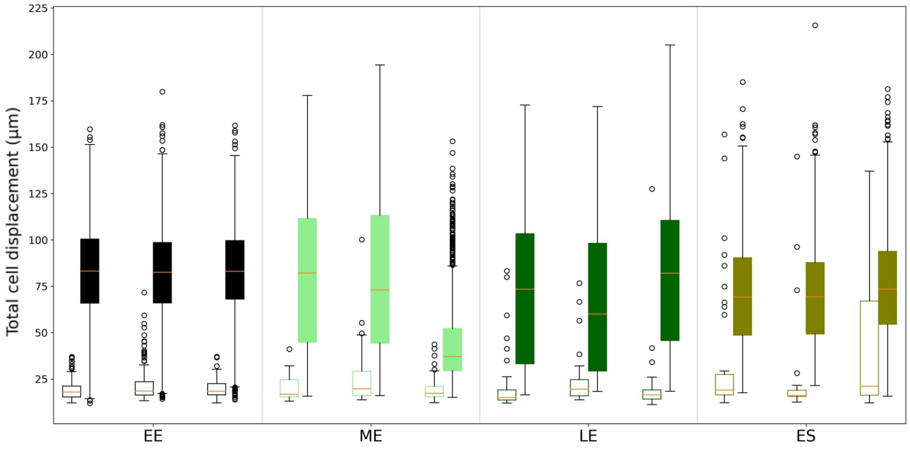

Figure S5: **Total cell displacement of cells harvested from populations in EE, ME, LE and ES phases (related to Fig. 5).** Cells total displacement during the time of the experiment (1h) measured for the non-migrating cells class (empty boxes) and the migrating cells class (filled boxes) for populations harvested in EE, ME, LE and ES phase. Total displacement of non-migrating cells is significantly lower than that of migrating cells in every populations (Mann–Whitney U test, p-values < 0.0005). No significant variation in total displacement of migrating cells was observed between populations harvested in different growth phases (mixed effects model ANOVA, p-value=0.4691).

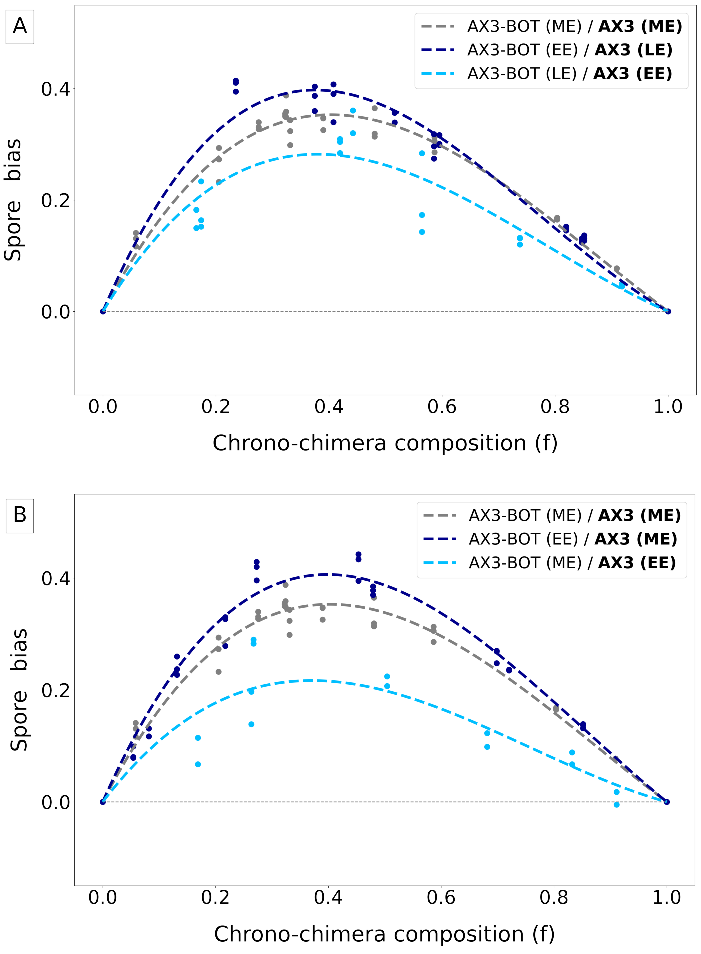

Figure S6: **Spore bias measured in chrono-chimeras with a lineage selected for increased adhesion (related to Fig. 6)**. Aggregation was initiated by mixing a strain that was obtained through experimental evolution for higher adhesiveness to the substratum (AX3-Bottom,[?]) with its ancestor (AX3). Three different combinations of growth phases are considered (as indicated in the legend). **A:** Strains are grown in co-culture and harvested in ME phase (gray line), the focal population is harvested in EE phase and the other in ME phase (dark blue line), or vice-versa (light blue line). **B:** Strains are grown in co-culture and harvested in ME phase (gray line), the focal population is harvested in EE phase and the other in LE phase (dark blue line), or vice-versa (light blue line). Spore bias variations are consistent whether the more advanced population is harvested in middle or late exponential phase, as it was observed for isogenic strains (Fig. 2), but starting from a high baseline bias.

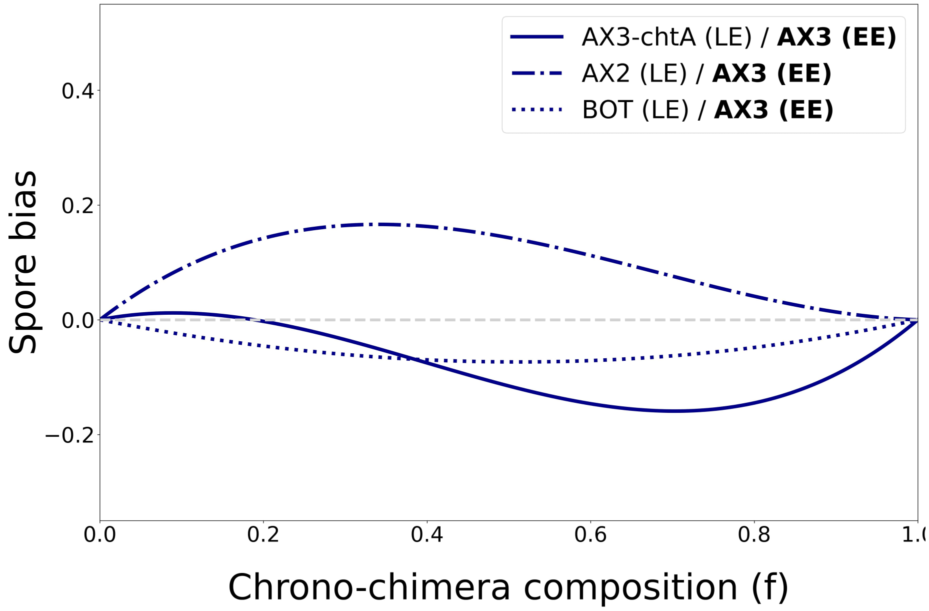

Figure S7: **Contribution of growth phase differences to spore bias depends on genetic background (reated to Fig. 6)**. Comparison of spore bias when the focal population AX3 (EE) is mixed with different strains in late exponential phase (LE). For every chimera, the curve indicates the deviation of the fitted spore bias from that for reference chimeras, where the two strains were harvested in the same growth phase (ME). The qualitative effect of growth phase differences is not consistent across the different chrono-chimeras, suggesting that it does not simply add up to variation induced by genetic differences, but instead depends on the genetic background.
